# Multi-omics evidence reveals a causal role of endoplasmic reticulum stress in cancer development

**DOI:** 10.7717/peerj.21164

**Published:** 2026-04-24

**Authors:** Rui Guo, Xunan Qiu, Tingting Tao, Yingying Wang, Shuwen Zheng, Siru Nie, Qingyue Zhang, Yuehua Gong

**Affiliations:** 1 Tumor Etiology and Screening Department of Cancer Institute and General Surgery, First Hospital of China Medical University, Shenyang, China; 2 Key Laboratory of Cancer Etiology and Prevention in Liaoning Education Department, First Hospital of China Medical University, China; 3 Key Laboratory of GI Cancer Etiology and Prevention in Liaoning Province, First Hospital of China Medical University, Shenyang, China; 4 Department of Pathology, The First Hospital of China Medical University, Shenyang, Liaoning, China

**Keywords:** Endoplasmic reticulum stress, Cancer risk, Mendelian randomization

## Abstract

Endoplasmic reticulum stress (ERS) plays a crucial role in the pathogenesis of various diseases, but its causal involvement and therapeutic potential in cancer remain unclear. In this study, we integrated genome-wide association study (GWAS) data from 18 common cancers with quantitative trait loci (cis-eQTL, cis-mQTL, and cis-pQTL) to explore the causal effects of ERS-related genes on cancer. A total of 1,350 ERS-related genes were retrieved from the GeneCards database. Mendelian randomization (MR) and Bayesian colocalization analyses were conducted to assess causality and shared genetic variants across mRNA expression, DNA methylation, and protein expression levels. External datasets were used for expression validation and diagnostic efficacy assessment. To provide experimental evidence, immunohistochemical (IHC) staining was performed to verify the expression and localization of key ERS-related genes in tumor and adjacent normal tissues. Functional enrichment, cellular localization, and drug sensitivity analyses were further applied to reveal potential biological mechanisms. We identified nine ERS-related genes and 15 methylation sites with potential causal relationships to specific cancer types. Both external validation and IHC analysis consistently confirmed the associations of *CBY1*, *CASP8*, *PLOD1*, and several methylation sites (cg09907170, cg09395195, cg08129017, and cg14808739) with their corresponding cancers. This comprehensive multi-omics and experimental validation study provides evidence supporting a causal role of ERS in cancer development and offers new insights into its molecular regulation and therapeutic potential.

## What’s New?

Endoplasmic reticulum stress (ERS) is a crucial pathological and physiological factor in the occurrence of diseases. However, its function as a potential pathogenic factor for cancer remains unknown. We used Mendelian randomization (MR) to analyze genome-wide association study (GWAS) data of 18 common cancers, identifying the relationship between the expression of nine ERS-related genes and the methylation sites of 15 genes with the risk of cancer. Further validation, drug sensitivity analysis revealed potential mechanisms and treatment targets in carcinogenesis.

## Background

The endoplasmic reticulum (ER) is a crucial location for regulating cell homeostasis and performs essential roles in protein synthesis, metabolic balance, and cell signaling (Schwarz & Blower, 2016). Various adverse conditions, including oxidative stress, chronic inflammation, and the acidic tumor microenvironment, can disrupt ER homeostasis and lead to endoplasmic reticulum stress (ERS) (Peng et al., 2025). A hallmark feature of ERS is the accumulation of unfolded or misfolded proteins within the ER lumen (Urra et al., 2016). While transient ERS activates adaptive mechanisms to restore ER function, prolonged or excessive ERS can overwhelm these protective responses, ultimately triggering maladaptive cellular processes such as metabolic reprogramming, autophagy, and apoptosis (Chen & Cubillos-Ruiz, 2021). Therefore, elucidating the biological significance of ERS is critical for understanding disease pathogenesis and developing therapeutic strategies. Research has indicated that a considerable number of malignant tumors in humans have severe ERS (Hetz, Zhang & Kaufman, 2020). Continuous exposure to ERS can stimulate the growth of tumor cells, the formation of new blood vessels, and the spread of cancer to other parts of the body (Cubillos-Ruiz, Bettigole & Glimcher, 2017). For example, cysteine-rich with EGF-like domains 2 (*CRELD2*), an ERS-related gene belonging to the cysteine-rich epidermal growth factor-like domain protein family, has been reported to enhance tumor angiogenesis and cell invasiveness, contributing to the development and progression of cancers such as prostate and renal carcinoma (Tang et al., 2023). In addition, epigenetic mechanisms, particularly DNA methylation, can modulate the expression of ERS-related genes, including *PTPN2* (encoding tyrosine phosphatase non-receptor type 2), *HSPA5* (encoding the ER chaperone *GRP78*), and other stress-responsive proteins. These alterations further influence ERS signaling pathways and contribute to tumor progression (Baumeister et al., 2005; Cao et al., 2019; Zhang et al., 2023b). ERS is additionally linked to tumor resistance against radiation and chemotherapy (Chang et al., 2020; Da Silva et al., 2020; Yao et al., 2020). It has been documented that in breast and colon cancer, ERS and the activation of associated molecules can alter the tumor cells’ chemoresistance to 5-Fluorouracil (5-FU) (Yao et al., 2020; Yun et al., 2017). Chemotherapy sensitivity may be increased by focusing on ERS-related signals, as supported by stress-amplifying anticancer strategies that induce excessive ROS production and DNA damage (Cao et al., 2021; Cao et al., 2023; Wang et al., 2022). Despite numerous studies demonstrating an association between ERS and tumorigenesis, further elucidation is required to establish a potential causal relationship between ERS and tumors. The precise pathogenic mechanism underlying ERS and its related molecules in tumorigenesis and targeted treatment strategies remain challenging. Mendelian randomization (MR) is a genetic epidemiological approach that utilizes genetic variants as instrumental variables (IVs) to establish causal relationships between exposure and outcome. This methodology aids in minimizing the impact of potential confounders and inverse associations, which has become widely employed in investigating the association between various genes and diseases (Chen et al., 2021; Davies, Holmes & Davey Smith, 2018). For instance, summary-based Mendelian randomization (SMR) and two sample mendelian randomization (TSMR) integrate GWAS data with gene expression, DNA methylation, and protein expression quantitative trait loci (eQTL, mQTL, and pQTL) to explore potential pathogenic variants in mitochondrial and oxidative stress-related genes (Li et al., 2023; Xu et al., 2023). However, a comprehensive causal evaluation of ERS-related genes across multiple molecular layers in human cancers is still lacking. This study employed a multi-omics MR framework for the first time to systematically evaluate the causal effects of 1,350 ERS-related genes at the transcriptional, methylation, and protein levels across 18 common cancers. By integrating SMR, HEIDI tests, Bayesian colocalization, and experimental validation in solid tumors, we aimed to identify key ERS driver genes with robust causal evidence and elucidate their underlying molecular mechanisms in specific cancer types.

## Methods

We used the Strengthening the Reporting of Observational Studies in Epidemiology (STROBE) guideline (Table S1) to structure this study.

### Data sources

**Figure 1:**
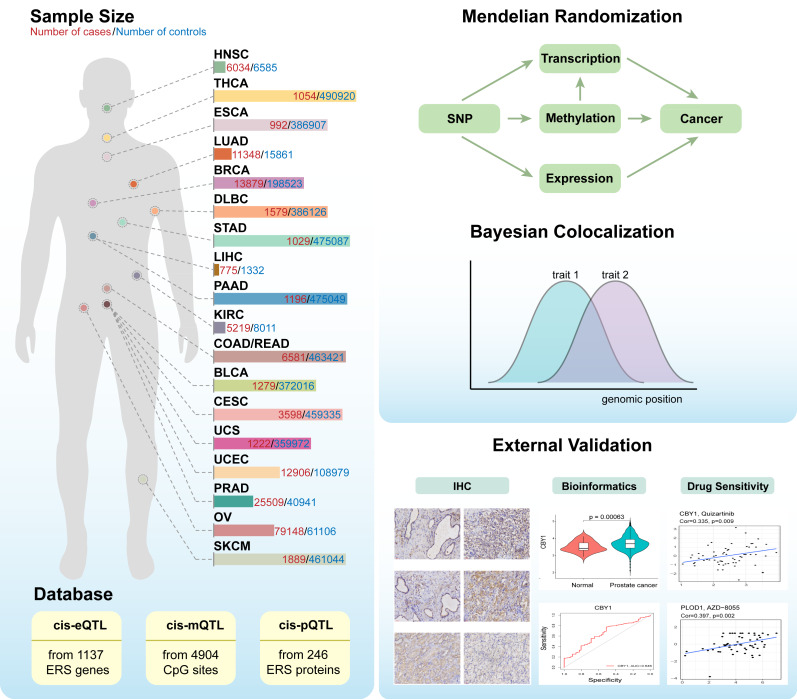
The design of this study and the workflow of the genetic variation selection and analysis methods.

GeneCards (https://www.genecards.org/) was used to extract 1,350 ERS-related genes, and genes with a relevance score greater than five were selected. Multiple large genome-wide association databases were used for our MR analyses (Table S2). The genetic variants strongly associated with the expression of ERS-related genes were extracted from the aggregated eQTL statistics of the eQTLGen consortium (https://www.eqtlgen.org/cis-eqtls.html) and were utilized as instrumental variables for gene expression, specifically focusing on cis-genetic variants within a 1,000 kb range on either side of the coding sequence (Võsa et al., 2021). The MR cis-mQTL instrument, which captures genetic variants strongly associated with methylation patterns in ERS-related genes, was derived from pooled data obtained through meta-analyses of two cohorts comprising a total of 1,980 individuals (Wu et al., 2018). The MR cis-pQTL instrument was developed based on the Icelandic proteome dataset, which encompasses genome-wide association data for 4,907 proteins, to investigate genetic variations in the expression of ERS-related proteins (Ferkingstad et al., 2021). The GWAS data for the 18 tumors in this investigation were gathered from publicly available databases (Table S2). Figure 1 shows a summary graphic of the experimental approach followed in this investigation.

### SMR and HEIDI analyses

MR analysis uses single nucleotide polymorphisms (SNPs) to infer the causal relationship between exposure and outcome, and its theories and methods have been well developed (Davey Smith & Hemani, 2014; Ference et al., 2012; Smith & Ebrahim, 2003). The SMR analysis integrates the summary statistics of QTL studies and GWAS within the framework of MR to estimate the impact of gene expression and DNA methylation on phenotypes associated with common cancers (Zhu et al., 2016). This approach enables systematic prioritization of functionally relevant molecular traits whose genetically predicted levels may influence disease risk, thereby bridging genetic variation and downstream biological mechanisms. The SMR framework is specifically designed to handle linkage disequilibrium in genomic regions dense with variants, which is common in omics QTL studies, and the HEIDI test helps distinguish whether an association is driven by pleiotropy or linkage. Therefore, the combined use of SMR and HEIDI provides an initial screen for putative causal relationships while reducing false-positive findings driven by correlated but distinct variants. There are three primary steps involved in the initial SMR analysis: (1) SNPs as IVs, gene expression as exposure, and multiple cancers as outcomes; (2) SNPs as IVs, DNA methylation as exposure, and multiple cancers as outcomes; (3) SNPs as IVs, DNA methylation as exposure, gene expression as outcome. The third step of the analysis contained only the significant findings from the first and second steps.

The HEIDI test examined whether significant associations obtained through SMR could be attributed to pleiotropic or linkage models, where a single shared genetic variant or multiple independent genetic variants influence both exposure and outcome variables. This test aimed to assess the null hypothesis of a singular causal mechanism underlying the association (pleiotropy) (Giambartolomei et al., 2014). Only associations consistent with a pleiotropic model were retained, ensuring that subsequent analyses focused on loci with a higher likelihood of reflecting genuine biological causality.

SMR and HEIDI analyses were performed using SMR software (https://yanglab.westlake.edu.cn/software/smr/#Overview) version 1.3.1. *P*_HEIDI_ > 0.01 and false discovery rate (FDR) < 0.05 were the criteria used to identify the final putative causal relationships. The following formula was used to calculate the odds ratio (OR) of ERS to cancer risk as a result of SMR: OR = exp(*β*_SMR_), where *β*_SMR_ is the estimated effect size of methylation or gene expression on cancer, OR is the estimated odds ratio for each 1-ln increase in ERS-related genomic level, and exp is the base of the natural logarithm.

### Bayesian colocalization analysis

To assess whether two traits are consistent with a shared causal variant and to separate linkage disequilibrium from confounding, the “coloc” package was used for Bayesian colocalization analysis (Giambartolomei et al., 2014). This analysis was performed as an additional validation step to determine whether SMR-supported associations arise from a common underlying causal variant rather than from correlated but distinct variants in linkage disequilibrium. Five hypotheses are involved in colocalization analysis: (1) in the genomic locus, there was no causal variant for either trait (H0); (2) one causal variant for trait one only (H1); (3) one causal variant for trait two only (H2); (4) there were two distinct causal variants for both traits (H3); and (5) there was a shared causal variant for both traits (H4). Colocalization analysis was conducted for all SNPs within 1,000 kb upstream and downstream of each SNP in this study. Under different priors and windows, the posterior probability for H4 (PP.H4) is greater than 80%, regarded as compelling evidence of colocalization. By providing a Bayesian probabilistic framework that jointly models both traits, colocalization analysis offers complementary evidence to HEIDI testing and strengthens locus-level causal inference.

### TSMR analysis

For the analysis of protein-cancer associations, we employed a TSMR framework, which is the standard approach for causal inference using curated lists of instrumental variables such as the sentinel cis-pQTLs from the deCODE study. In this study, the TwoSampleMR R package was utilized to analyze pQTL data and GWAS data to investigate the causal associations between ERS-related genes and various types of cancer. For features containing only one SNP, the Wald ratio test was used to estimate the association between the identified SNPs and each cancer (Burgess, Small & Thompson, 2017). For features containing multiple SNPs, five commonly used MR methods were utilized: simple mode, inverse-variance weighted (IVW), MR-Egger, weighted median, and weighted mode. Given the superior validity of the IVW method, when multiple SNPs were utilized, our results primarily focused on the IVW method, complemented by the other four methods. To assess potential horizontal pleiotropy and heterogeneity, we employed the MR-Egger intercept test and Cochran’s *Q* statistic (both *P* > 0.05). Together, these sensitivity analyses enhance confidence that the observed associations are unlikely to be driven by pleiotropy or heterogeneity. The analyses for this section were conducted using R software (version 4.3.0, http://www.R-project.org).

### Phenotype scanning

The PhenoScanner database (https://github.com/phenoscanner/phenoscanner) explored the relationships between identified SNPs and other traits. The following were the screening requirements for phenotype scanning: (1) GWAS data originated from individuals of European ancestry; (2) the SNP exhibited genome-wide significance in its association with the trait (*P* < 5 × 10^−8^); and (3) SNPs were linked to any known disease risk factors.

### External validation and diagnostic efficacy evaluation

The mRNA expression and CpG site methylation data of critical genes in various types of cancers were collected from the TCGA database (https://cancergenome.nih.gov). The “ggpubr” and “ggplot2” R packages were used to compare the expression levels of critical genes and CpG site methylation between tumor and normal tissues while also analyzing the association between gene expression and CpG site methylation levels. The “pROC” package was also employed for Receiver Operating Characteristic (ROC) curve analysis to evaluate the diagnostic efficacy of crucial gene expression and CpG site methylation levels in tumors.

### Immunohistochemical staining

All human tissue specimens used in this study were obtained from the First Affiliated Hospital of China Medical University and approved by the Medical Ethics Research Committee of the First Affiliated Hospital of China Medical University (Ethical Application Ref: [2025]473). Informed consent has been waived for this study.

A total of twenty formalin-fixed, paraffin-embedded (FFPE) tissue specimens were collected, including ten cases of prostate adenocarcinoma and ten cases of renal cell carcinoma, each with matched adjacent non-neoplastic tissues. All specimens were obtained from the First Affiliated Hospital of China Medical University. Protein expression levels were evaluated by immunohistochemical (IHC) staining. Tissue sections were subjected to antigen retrieval in EDTA buffer, followed by incubation with primary antibodies against *CBY1*, *CASP8*, or *PLOD1* (Proteintech, 12239-1-AP, 13423-1-AP, 29480-1-AP). Two experienced pathologists independently evaluated the staining results in a double-blind manner. The staining intensity was graded on a four-point scale: 0 (negative), 1 (weak), 2 (moderate), and 3 (strong). The percentage of positively stained area was scored as follows: 0 (0–5%), 1 (6–25%), 2 (26–50%), 3 (51–75%), and 4 (76–100%). The final immunoreactivity score (IRS) was calculated as the product of the intensity and percentage scores.

### Cell type-specific enrichment and gene function analysis

TISCH2 (http://tisch.comp-genomics.org/; accessed 11 January 2025) is a single-cell mRNA-seq database housing an extensive collection of single-cell data and cell-type annotations for human cancers. Leveraging this database, we investigated the specific expression patterns of essential genes across different cancer cell types.

Gene function analysis was conducted using mRNA-seq data sourced from the TCGA database. Based on the median mRNA expression levels of these critical genes in various tumors, tumor samples were categorized into high and low-expression groups. Differential gene (DEG) expression analysis utilized the “limma” R package. All genes were ranked according to their logFoldChange (logFC) values obtained from this differential expression analysis. Subsequently, gene set enrichment analysis was conducted using the “clusterProfiler” package with KEGG gene sets, GO-BP gene sets, CancerSEA gene sets, Hallmark gene sets, Reactome gene sets, and wiki pathways gene sets as references. The top 5 enriched pathways in both high and low-expression groups were visualized.

### Drug sensitivity analysis of gene expression and CpG site methylation

mRNA-seq expression profiles and DNA methylation beta-values from the CellMiner database were downloaded to analyze the drug sensitivity of crucial gene expression and CpG site methylation (Shankavaram et al., 2009). Drugs approved by the FDA or clinical trials were selected for analysis using R version 4.3.0. The “limma”, “impute”, “ggplot2”, and “ggpubr” R packages were utilized to examine the relationship between gene expression, CpG site methylation level, and minor molecule drug sensitivity.

## Results

### Genetic IV selection

According to the cis-eQTL data from the eQTLGen database, SNPs related to 1,137 transcriptome expressions associated with ERS-related genes were selected. Additionally, we chose SNPs corresponding to 4,904 DNA methylation CpG sites of the genes associated with ERS based on McRae et al.’s study. From the deCODE database, 246 ERS-related protein SNPs were taken out. The screening criteria were that all SNPs included in the initial analysis had: (1) at least a suggestive *P* < 5 × 10^−8^; (2) an *F*-statistic ≥ 10; and (3) a minor allele frequency (MAF) >0.01. Therefore, SNPs with solid associations with ERS-related gene expression, DNA methylation, and protein expression were obtained for subsequent analysis.

### MR and colocalization analysis for the association between the expression of ERS-related genes and multiple cancer outcomes

The relationship between SNPs representing ERS-related gene expression and cancer outcomes following SMR is illustrated in Table S3. By considering adjusted *P* values (FDR < 0.05) and conducting the HEIDI test (*p* _HEIDI > 0.01), a total of 35 genes across nine cancer types (35 significant gene-trait associations) were identified. No significant association was found between ERS-related gene expression and other types of cancers.

**Figure 2:**
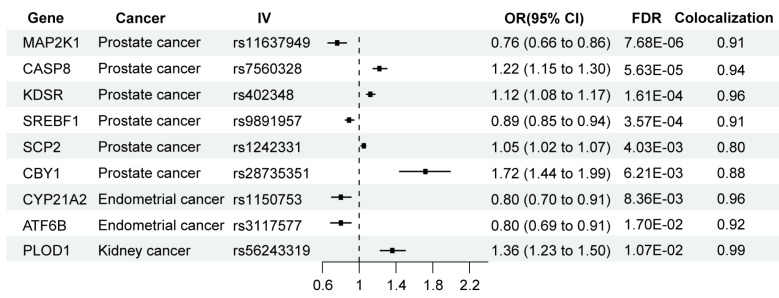
MR and colocalization results for the association between the expression of ERS-related genes and cancer outcomes. IV, Instrumental variant; OR, Odds ratio; 95% CI, 95% Confidence Interval. The values in the colocation column represent PP.H4 between eQTL and cancer outcomes. PP.H4 > 0.8 is the well-applied cut-off for the evidence of colocalization.

Further Bayesian colocalization analysis revealed that nine genes shared causal variants in their expression patterns related to three types of cancers, which are *MAP2K1* (PP.H4 = 0.91), *CASP8* (PP.H4 = 0.94), *KDSR* (PP.H4 = 0.96), *SREBF1* (PP.H4 = 0.91), *SCP2* (PP.H4 = 0.80) and *CBY1* (PP.H4 = 0.88) in prostate cancer; *CYP21A2* (PP.H4 = 0.96) and *ATF6B* (PP.H4 = 0.92) in endometrial cancer; and *PLOD1* (PP.H4 = 0.99) in kidney cancer (Fig. 2). Among them, five genes were found to have higher expression levels that are linked to a higher risk of cancer, including *CASP8* (OR: 1.22, 95% CI [1.15–1.30], FDR = 5.63 × 10^−5^), *KDSR* (OR: 1.12, 95% CI [1.08–1.17], FDR = 1.61 × 10^−4^), *SCP2* (OR: 1.05, 95% CI [1.02–1.07], FDR = 4.03 × 10^−3^) and *CBY1* (OR: 1.72, 95% CI [1.44–1.99], FDR = 6.21 × 10^−3^) in prostate cancer; *PLOD1* (OR: 1.36,95% CI [1.23–1.50], FDR = 1.07 × 10^−2^) in kidney cancer.

### MR and colocalization analysis for the association between CpG site methylation of ERS-related genes and multiple cancer outcomes

After applying FDR correction and conducting the HEIDI test (Table S4), we identified a total of 18 unique genetic loci associated with 24 signals in breast cancer, three unique genetic loci related to three signals in cervical cancer, and 13 unique genetic loci associated with 15 signals in endometrial cancer. Additionally, we found that lung cancer had associations with 15 unique genetic loci for 18 signals, ovarian cancer had associations with 26 unique genetic loci for 33 signals, and prostate cancer had associations with 96 unique genetic loci for a total of 133 signals. In comparison, gastric cancer showed associations with six unique genetic loci for 11 signals; thyroid cancer showed one signal. No significant genetic associations were observed between DNA methylation of ERS-related genes and other types of cancers.

**Figure 3:**
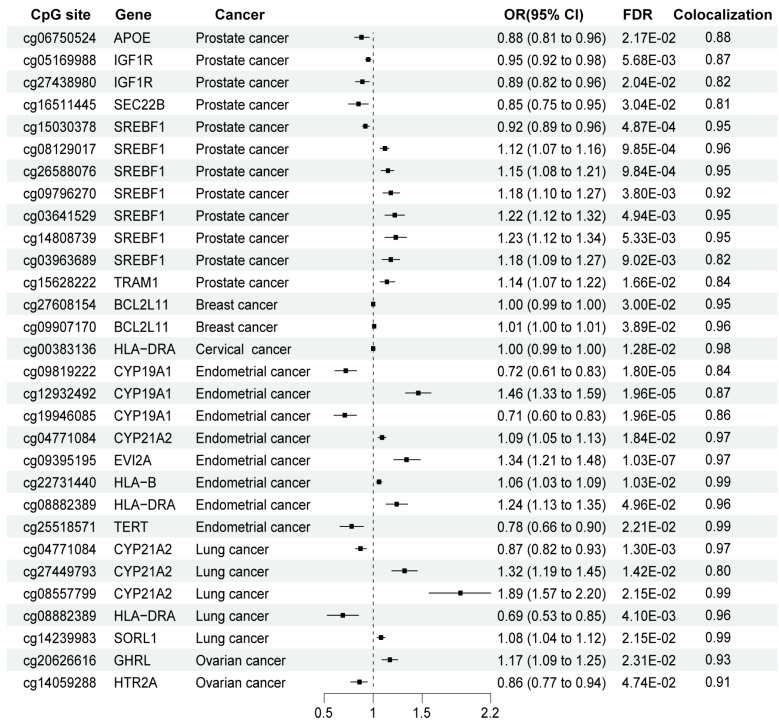
MR and colocalization results for the association between CpG site methylation of ERS-related genes and cancer outcomes. OR, Odds ratio; 95% CI, 95% Confidence Interval. ‘Colocalization’ indicates PP.H4 between mQTL and cancer outcomes. PP.H4 > 0.8 is the well-applied cut-off for the evidence of colocalization.

Bayesian colocalization revealed that 28 CpG site methylation of 15 genes shared causal variants with six types of cancers (Table S5 and Fig. 3). Among them, different methylation sites within the same gene may exert distinct effects on the disease outcomes. For example, in prostate cancer, the methylation levels of six out of the seven CpG sites in the *SREBF1* gene were found to be positively associated with prostate cancer risk. In contrast, one site showed a negative association. In lung cancer, among the three CpG sites of the *CYP21A2* gene, the methylation level of the two sites was positively correlated with the risk of lung cancer. In endometrial cancer, the methylation status of one CpG site in the *CYP19A1* gene was positively correlated with endometrial cancer risk among the three sites examined. In breast cancer, the methylation levels of two distinct CpG sites in *BCL2L11* exhibited positive and negative associations with the risk of developing breast cancer, respectively.

### Integrated analysis of gene expression and CpG site methylation of ERS-related genes

The altered DNA methylation of genes has been reported to impact their mRNA expression (Weisenberger, 2014). Therefore, we conducted SMR analysis to investigate the causal relationship between CpG site methylation and the expression of ERS-related genes by mapping methylation to expression through shared genetic variants. Following FDR correction and HEIDI test, we obtained 59 genes, including *TERT*, *AKT1*, *ESR1*, *NOTCH1*, *SREBF1*, and many other genes, whose expression was controlled by DNA methylated CpG sites (Table S6).

### MR and colocalization analysis for the association between protein expression of ERS-related genes and multiple cancer outcomes

**Figure 4:**
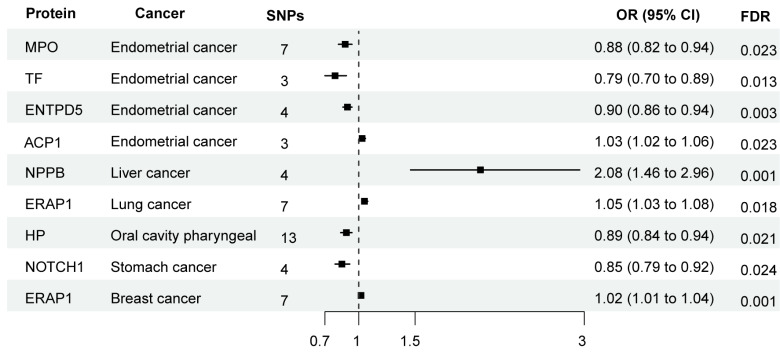
MR-IVW and sensitivity analyses of the association between the protein expression of ERS-related genes and cancer outcomes. OR, Odds Ratio; 95% CI, 95% Confidence Interval.

The causal relationship between the protein expression of ERS-related genes and various cancer outcomes was analyzed using five MR methods, primarily the IVW method. Additionally, heterogeneity and pleiotropy tests were conducted, along with Bayesian colocalization analysis. Our findings suggested that *MPO*, *TF*, *ENTPD5*, *ACP1*, *NPPB*, *ERAP1*, *HP*, and *NOTCH1* genes were associated with the risk of corresponding cancer (Fig. 4 and Table S7). However, there was no evidence of shared genetic variation between these genes and corresponding cancer (Table S8).

### Phenome-wide scan of genetic variants

To exclude the possibility of pleiotropy in the studied cancers, we conducted a phenome-wide scan analysis of the genetic variants obtained from the colocalization analyses described above using the PhenoScanner database. The results are presented in Table S9. Interestingly, genetic variants causally correlated with the gene expression of *KDSR* and *CBY1* in prostate cancer, and *CYP21A2* and *ATF6B* gene expression in endometrial cancer were not found to be linked to all available minor traits.

Among CpG site methylation-associated genetic variants derived from the colocalization analyses, the associations of rs4598682 (*SORL1*-methylation related) in lung cancer, rs27647 (*GHRL*-methylation related) and rs9534509 (*HTR2A*-methylation related) in ovarian cancer, rs76051950 (*SEC22B*-methylation related) and rs111745460 (*TRAM1*-methylation related) in prostate cancer with potential secondary traits were not observed. It is worth noting that some SNPs linked to gene methylation showed associations with multiple characteristics. For instance, the association between rs769449 (*APOE* methylation-related) in prostate cancer was also observed for metabolism, height, and weight. These findings suggest a certain level of robustness in the identified causal relationships; however, it should be acknowledged that genetic variants associated with minor traits may introduce horizontal pleiotropy, which requires further investigation to rule out.

### External validation and diagnostic efficacy evaluation of gene expression and CpG site methylation

**Figure 5:**
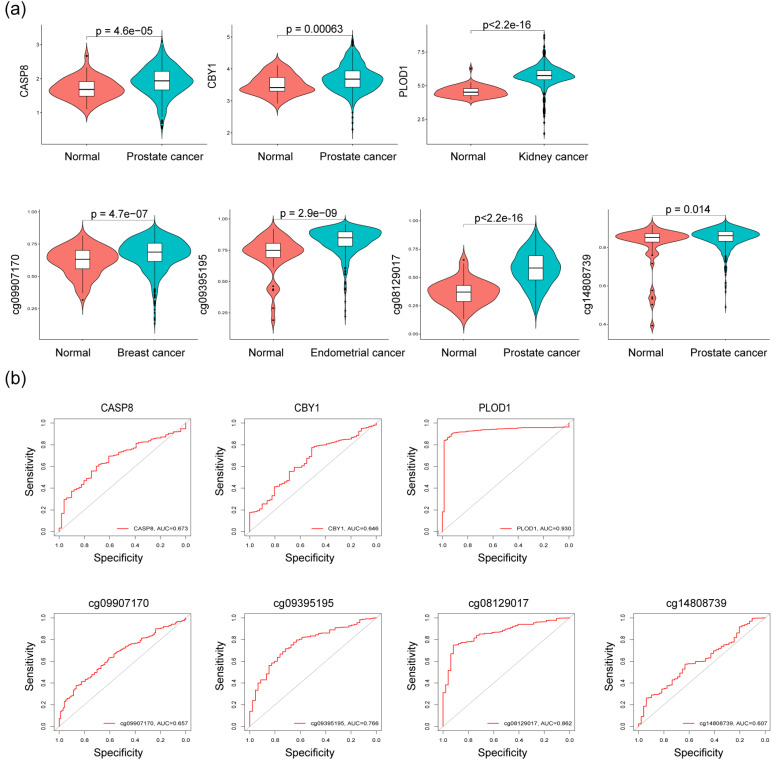
External validation and diagnostic efficacy evaluation. (A) The expression levels of CBY1, CASP8, PLOD1 and the methylation levels of cg09907170, cg09395195, cg08129017, and cg14808739 in corresponding tumors. (B) ROC curve analysis of CBY1, CASP8, PLOD1, cg09907170, cg09395195, cg08129017, cg14808739 in corresponding tumors.

To confirm the prioritized genes and CpG sites obtained from the colocalization analyses, we initially validated their differential expression using mRNA-seq and methylation data from the TCGA database in both normal and cancer samples (Fig. 5). The results of our analysis on DEG expression revealed that *CASP8* and *CBY1* exhibited high expression levels in prostate cancer, while *PLOD1* showed elevated expression in kidney renal clear cell carcinoma. Furthermore, immunohistochemical analysis of paraffin-embedded tissue specimens obtained from patients at the First Affiliated Hospital of China Medical University demonstrated that, compared with adjacent normal tissues, both *CASP8* and *CBY1* were significantly overexpressed in prostate cancer tissues (*P* < 0.05), whereas *PLOD1* was markedly downregulated in renal cell carcinoma tissues (*P* < 0.001) (Fig. S1). These findings were consistent with and provided supportive evidence for the aforementioned MR analysis, suggesting that increased gene expression is associated with an augmented risk of developing cancer. Differential analysis of CpG site methylation levels revealed that, corresponding to the above MR Analysis results, the cg09907170 site of the *BCL2L11* gene in breast cancer, the cg09395195 site of the *EVI2A* gene in endometrial cancer, and the cg08129017 and cg14808739 sites of the *SREBF1* gene in prostate cancer exhibited elevated methylation levels associated with a higher risk for their respective cancers.

In addition, the ROC diagnostic performance evaluation results indicated that the validated differences in gene expression and methylation levels were associated with diagnostic values for their respective cancers. Specifically, *PLOD1*, cg09395195, and cg08129017 demonstrated an area under the ROC curve (AUC) greater than 0.75. The AUC values for *CASP8*, *CBY1*, cg09907170, and cg14808739 were 0.673, 0.646, 0.657, and 0.607, respectively. Due to data limitations, the remaining genes and loci that were not detected or showed inconsistency with the results of the MR analysis were not listed and were excluded from further analysis.

### Cell type-specific enrichment and gene function analysis

For the above genes that matched the MR results in the TCGA database, we performed a cell type-specific analysis using the TISCH2 single-cell database (Fig. S2). The results showed that *CBY1* was mainly expressed in epithelial and endothelial cells, and *CASP8* was expressed primarily in T cells and NK cells in prostate cancer. *PLOD1* was mainly expressed in endothelial cells in kidney renal clear cell carcinoma.

Further functional enrichment analysis showed that, in prostate cancer, *CBY1* was mainly related to intracellular trafficking and oxidative phosphorylation pathways, while *CASP8* was mainly related to cell cycle and immune response pathways. *PLOD1* was associated with epithelial-mesenchymal transition in renal clear cell carcinoma (Fig. S3).

### Drug sensitivity analysis of gene expression and CpG site methylation

**Figure 6:**
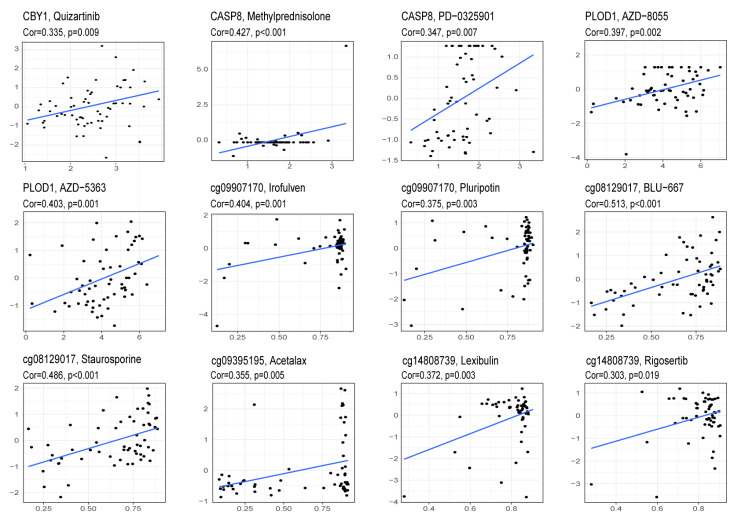
Drug sensitivity analysis of the expression of CBY1, CASP8, PLOD1 genes and the methylation of cg09907170, cg09395195, cg08129017, cg14808739 CpG sites.

Increased drug sensitivity is critical to prevent cancer cells from developing resistance to treatment. To explore this further, we utilized data from the CellMiner database to analyze the association between drug sensitivity and the expression levels of genes, as well as the methylation levels of CpG sites obtained from the validation above. The results demonstrated a significant positive association between *CBY1* and the drug sensitivity to Quizartinib. *CASP8* exhibited positive associations with the sensitivities of Methylprednisolone and PD-0325901. *PLOD1* displayed positive associations with the sensitivities of AZD-5363 and AZD-8055. A noteworthy positive association was observed between cg09907170 and the sensitivities of Irofulven and Pluripotin. cg09395195 exhibited a significant positive association with Acetalax, while cg08129017 showed significant positive associations with BLU-667 and Staurosporine. Lastly, cg14808739 demonstrated a significant positive association with the sensitivities of Lexibulin and Rigosertib. The findings suggest a complex interplay between gene expression, CpG site methylation levels, and drug sensitivity across diverse cancer types, offering promising targets for therapeutic intervention in oncology (Fig. 6).

## Discussion

In this study, we systematically analyzed the causal relationship between ERS-related genes and the risk of multiple cancers. Following SMR and colocalization analysis, nine genes’ expression and 15 genes’ methylation patterns at multiple CpG sites were identified as showing genetic evidence supportive of an association with cancer risk. Further external validation revealed that three critical genes (*CBY1*, *CASP8*, *PLOD1*) and four key CpG methylation sites (cg09907170, cg09395195, cg08129017, cg14808739) were differently expressed in the corresponding cancers. Furthermore, cellular localization and functional analysis, as well as drug sensitivity studies, were carried out. Our findings shed light on potential underlying molecular mechanisms of ERS in carcinogenesis and identify key candidates for further investigation as treatment targets.

*CBY1*, traditionally characterized as a *β*-catenin antagonist and tumor suppressor in other contexts (Schuierer et al., 2006; Takemaru et al., 2003; Wen et al., 2021), was identified here as being potentially causally linked to prostate cancer risk. Beyond its canonical Wnt-regulatory role, emerging evidence suggests a functional crosstalk between the ERS/UPR pathway and Wnt/*β*-catenin signaling (Zhang et al., 2023a). In specific cell lines (such as leukemia cells), the expression of *CBY1* is regulated by ERS, and in turn, it affects the cell fate decision (survival or apoptosis) mediated by UPR (Mancini et al., 2013). Therefore, our genetic finding invites the hypothesis that *CBY1*’s role in prostate cancer may extend beyond Wnt suppression to involve the integration of ERS signals with oncogenic growth pathways. The association between high *CBY1* expression and sensitivity to Quizartinib further suggests a potential therapeutic vulnerability that may be linked to this stress-adapted cellular state (Erba et al., 2023), warranting future investigation. Further external validation and ROC analysis have provided evidence supporting *CBY1* as a potential therapeutic target in prostate cancer. As a member of the caspase family, *CASP8* plays a pivotal role in inflammation and cell survival, and traditionally regarded as the initiator of extrinsic apoptosis (Fulda, 2009; Shalini et al., 2015). However, recent studies have shown that in the ERS state, CASP8 is recruited to the autophagosome membrane to form an iDISC (intracellular death-inducing signaling complex), which is an atypical activation pathway that is not dependent on death receptors and is specifically triggered by ER stress. This explains why ERS-related gene screening enriches for CASP8 (Hattori et al., 2024). Studies have indicated that elevated expression of *CASP8* may be associated with an increased risk of prostate cancer recurrence (Liu et al., 2021). Our analysis further suggested that the upregulation of *CASP8* could potentially contribute to the risk of prostate cancer. Furthermore, external validation demonstrated that *CASP8* exhibited high expression levels in prostate cancer and displayed specific diagnostic values. Moreover, *CASP8* was predominantly expressed in T cells, implicating its involvement in immune response pathways corresponding to its cellular localization, consistent with previous reports (Kawadler et al., 2008). It is noteworthy that while Methylprednisolone and PD-0325901, the drugs mentioned above as highly sensitive to *CASP8*, have been utilized in prostate cancer treatment (Gioeli et al., 2011; Kassi & Moutsatsou, 2011), the potential targeting relationship between these drugs and *CASP8* has not been reported and requires further investigation. Procollagen-Lysine, 2-Oxoglutarate 5-Dioxygenase 1(PLOD1) is a key enzyme that catalyzes the hydroxylation of lysine residues in collagen. Predominantly localized in the endoplasmic reticulum, PLOD1 not only plays a critical role in collagen maturation and stabilization of the tumor extracellular matrix, but its dysfunction may also increase ER protein-folding burden and thereby trigger activation of the unfolded protein response UPR (Ishikawa et al., 2020; Lim et al., 2019). Mutations and aberrant overexpression of *PLOD1* have been implicated in the initiation and metastasis of various malignancies (Qi & Xu, 2018). However, its specific involvement in renal cancer remains unclear. In the present study, MR and TCGA analyses indicated that high *PLOD1* expression was associated with an increased risk of renal cell carcinoma, and the rs56243319 SNP may represent a potential susceptibility locus for this cancer. Interestingly, immunohistochemical validation revealed a markedly reduced expression of *PLOD1* protein in renal cell carcinoma tissues compared with adjacent normal tissues, which appears to contradict the results of our genetic and transcriptomic analyses. Furthermore, previous studies have indicated that *PLOD1* can facilitate cancer progression by inducing epithelial-mesenchymal transition in glioma and breast cancer (Song et al., 2017; Wang et al., 2021). Our integrative analysis supports the hypothesis that *PLOD1* participates in renal cell carcinoma pathogenesis through multifaceted regulatory pathways, and that its regulation may differ between genetic and protein levels. In addition, our drug sensitivity analysis identified AZD-5363 and AZD-8055 as potential compounds targeting *PLOD1*-associated pathways, offering new insights for the development of precision therapies in renal cell carcinoma. As a significant epigenetic modification, DNA methylation has garnered considerable attention due to its association with various diseases. In our study, by integrating the mQTL data of ERS-related genes and the GWAS data of 18 cancers, we identified 15 ERS-related genes whose methylation at multiple CpG sites was causally associated with cancer by SMR and colocalization analysis. Further external validation demonstrated that, in addition to the CpG sites that were either undetected or showed no difference between the normal and cancer groups, specific CpG sites (cg09907170, cg09395195, cg08129017, and cg14808739) exhibited high expression levels in breast cancer, endometrial cancer, and prostate cancer. These CpG sites also displayed specific diagnostic efficacy. This validation result aligns with and provides supportive evidence for the aforementioned MR result, indicating that increased methylation levels at these four CpG sites are associated with an elevated risk of developing their corresponding cancers. Moreover, drug sensitivity analysis provided valuable insights for targeted therapy strategies aimed at these specific methylation sites. Our findings offer further evidence and impetus for the role of DNA methylation in cancer.

In addition, we identified 59 ERS-related genes (*TERT*, *AKT1*, *ESR1*, *NOTCH1*, *SREBF1*, *etc*.) that could be affected by their methylation status. For the above genes, the regulation of gene expression by methylation in the promoter region has been mostly reported. For example, regulating *TERT* expression by methylation in its promoter region has been widely concerned and reported (Lee et al., 2020). Moreover, the medicine decitabine reduced the methylation of *TERT* promoter region (Lee et al., 2022). Therefore, the DNA methylation sites obtained in this study provide new clues for regulating DNA methylation on the expression of these genes.

Finally, it is worth mentioning that this study employed comprehensive MR and multi-omics approaches to investigate the causal association and potential therapeutic targets between ERS-related genes and various cancers. By incorporating large sample size and GWAS datasets, potential biases and confounding factors were effectively mitigated. Nevertheless, several limitations should be acknowledged. First, while the primary studies from which we sourced summary statistics (eQTL, mQTL, pQTL, and GWAS) adjusted for key covariates such as age, sex, and batch effects in their respective designs, the specific sets of covariates adjusted for were not uniform across all datasets. Although this heterogeneity is common in analyses leveraging publicly available summary data, it represents a potential source of residual confounding that should be considered when interpreting the causal estimates. In addition, although MR analysis can provide evidence for potential causal relationships, and some gene expression and methylation sites have been validated by external data, the limited sample size of the dataset still leaves many crucial pieces of information unexplored. Further experimental verification is essential to determining the final risk factors and the mechanism of drug targeting.

## Supplemental Information

10.7717/peerj.21164/supp-1 Supplemental Information 1 Immunohistochemical analysis of CASP8, CBY1, and PLOD1 expression in human cancer tissues (A) Immunohistochemical staining images (40X) and statistical analysis of CASP8 in prostate cancer and adjacent non-cancerous tissue; (B) Immunohistochemical staining images (40X) and statistical analysis of CBY1 in prostate cancer and adjacent non-cancerous tissue; (C) Immunohistochemical staining images (40X) and statistical analysis of PLOD1 in renal cell carcinoma and adjacent non-cancerous tissue. ^∗^*P* < 0.05; ^∗∗∗^*P* < 0.001.

10.7717/peerj.21164/supp-2 Supplemental Information 2 Cell type-specific enrichment at the single-cell level based on the TISCH2 database (A) Cell type-specific enrichment of CBY1 in prostate cancer. (B) Cell type-specific enrichment of CASP8 in prostate cancer. (C) Cell type-specific enrichment of PLOD1 in renal carcinoma.

10.7717/peerj.21164/supp-3 Supplemental Information 3 Function analysis of CBY1, CASP8, and PLOD1 (A) Function analysis of CBY1 in prostate cancer. (B) Function analysis of CASP8 in prostate cancer. (C) Function analysis of PLOD1 in renal carcinoma .

10.7717/peerj.21164/supp-4 Supplemental Information 4 Supplementary tables

10.7717/peerj.21164/supp-5 Supplemental Information 5 Raw data for the IHC score analysis

10.7717/peerj.21164/supp-6 Supplemental Information 6 The codes for SMR and HEIDI analyses

10.7717/peerj.21164/supp-7 Supplemental Information 7 The code for TSMR analysis

10.7717/peerj.21164/supp-8 Supplemental Information 8 The code for colocalization analysis

10.7717/peerj.21164/supp-9 Supplemental Information 9 The code for ROC curve

10.7717/peerj.21164/supp-10 Supplemental Information 10 The code for drug sensitivity

## Additional Information and Declarations

### Data Availability

The following information was supplied regarding data availability: The raw data for the IHC score analysis and code are available in the Supplemental Files.
