# Supplementary figures and images for: Multi-omics evidence reveals a causal role of endoplasmic reticulum stress in cancer development
Source: PeerJ. 2026 Apr 24;14:e21164. doi: 10.7717/peerj.21164 (PMC13116417; doi:10.7717/peerj.21164)

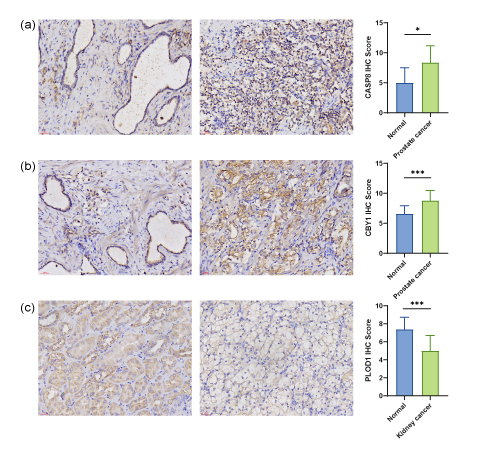

Supplement: Supplemental Information 1 — (A) Immunohistochemical staining images (40X) and statistical analysis of CASP8 in prostate cancer and adjacent non-cancerous tissue; (B) Immunohistochemical staining images (40X) and statistical analysis of CBY1 in prostate cancer and adjacent non-cancerous tissue; (C) Immunohistochemical staining images (40X) and statistical analysis of PLOD1 in renal cell carcinoma and adjacent non-cancerous tissue. ∗P < 0.05; ∗∗∗P < 0.001. [file peerj-14-21164-s001.tif]

(a)

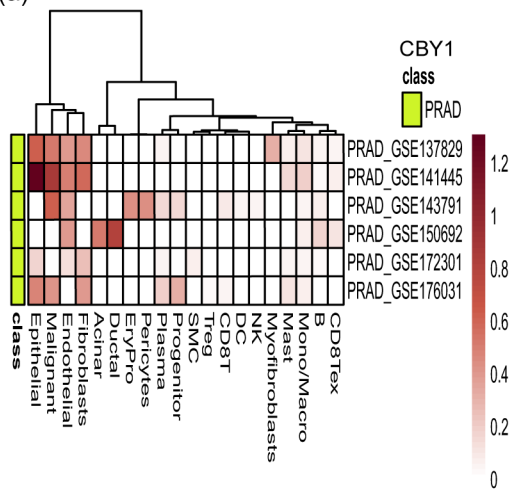

(b)

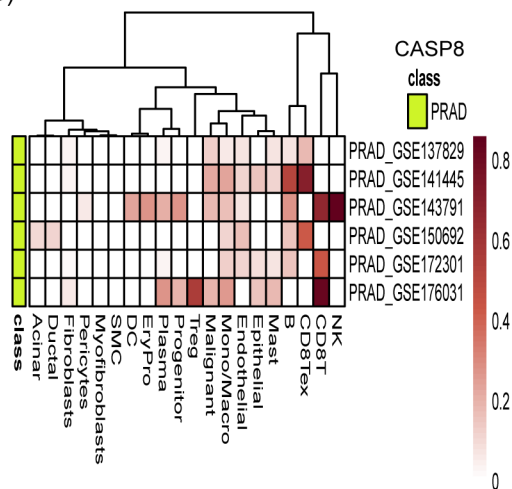

(c)

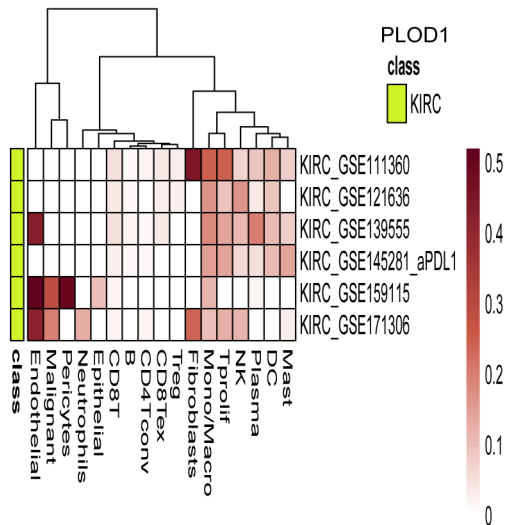

Supplement: Supplemental Information 2 — (A) Cell type-specific enrichment of CBY1 in prostate cancer. (B) Cell type-specific enrichment of CASP8 in prostate cancer. (C) Cell type-specific enrichment of PLOD1 in renal carcinoma. [file peerj-14-21164-s002.pdf]

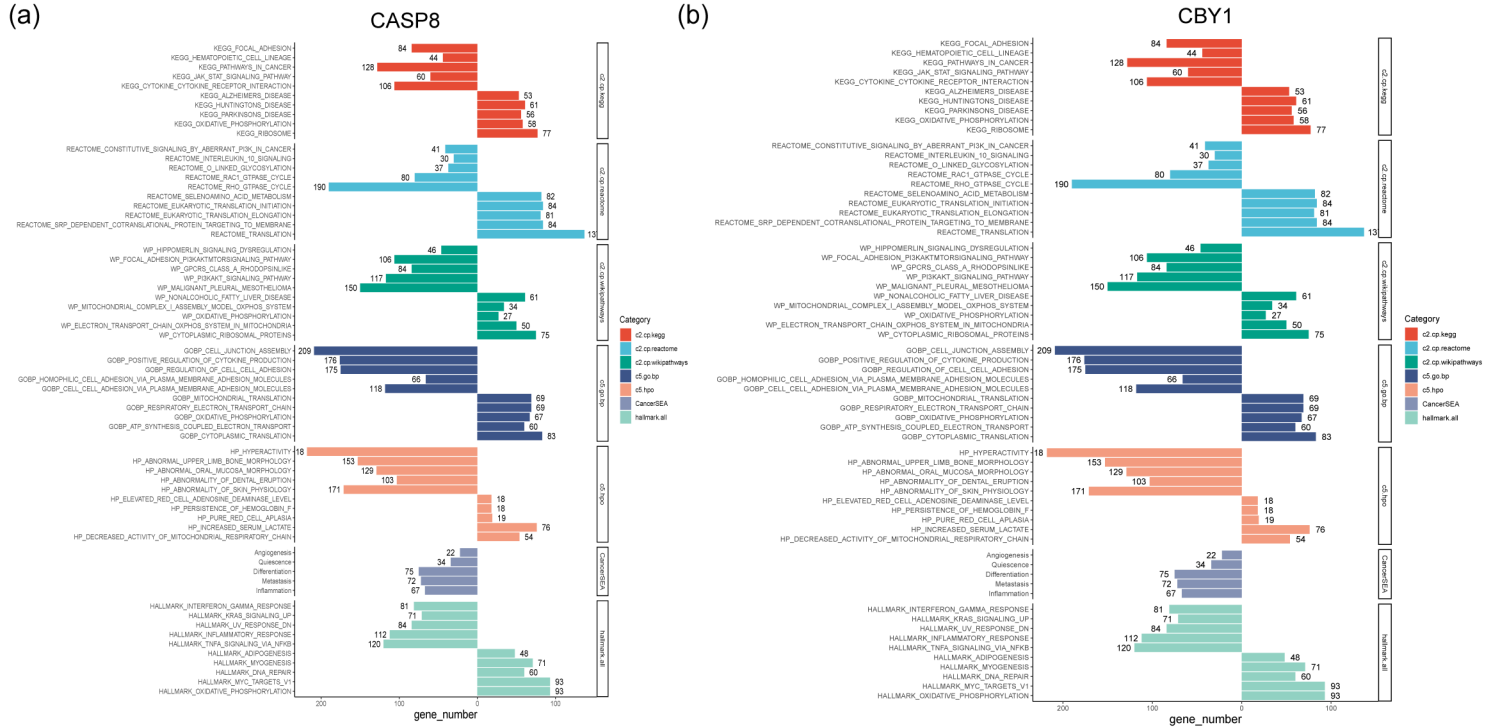

(c)

PLOD1

Supplement: Supplemental Information 3 — (A) Function analysis of CBY1 in prostate cancer. (B) Function analysis of CASP8 in prostate cancer. (C) Function analysis of PLOD1 in renal carcinoma . [file peerj-14-21164-s003.pdf]
